# Supplementary figures and images for: Variation in the serotonin transporter genotype is associated with maternal restraint and rejection of infants: A nonhuman primate (Macaca mulatta) model
Source: PLoS One. 2023 Apr 24;18(4):e0281935. doi: 10.1371/journal.pone.0281935 (PMC10124887; doi:10.1371/journal.pone.0281935)

## Effects of Maternal 5-HTT Genotype on the Frequency of Maternal Rejections of Infants

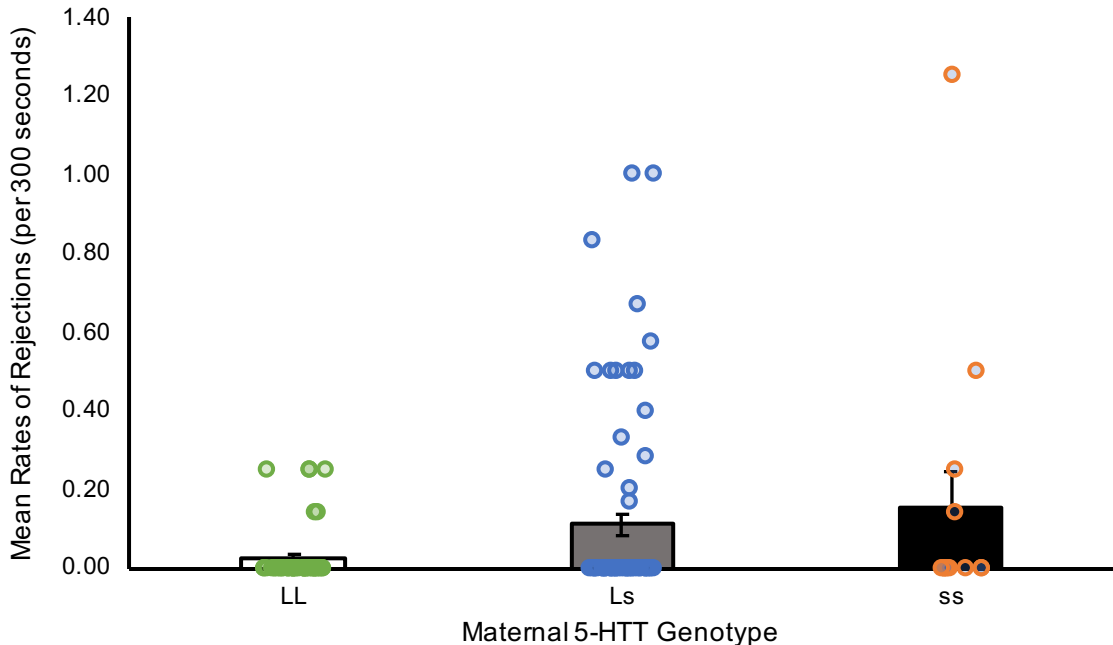

Supplement: S1 Fig — Plot depicts jittered individual maternal rejection data points, grouped by maternal 5-HTT genotype. When compared to mothers that were homozygous for the L allele, mothers that were homozygous for the s allele exhibited higher rates of infant rejections (p = .03). Mothers that were homozygous for the L allele also exhibited fewer rejections, when compared to mothers that were heterozygous (p = .03). White bars/green data points indicate mothers that were homozygous for the L allele, gray bars/blue data points indicate heterozygous mothers, and black bars/orange data points indicate mothers that were homozygous for the s allele. Data points are jittered to increase visibility. Error bars are standard errors. (PDF) [file pone.0281935.s001.pdf]

# Effects of Maternal 5-HTT Genotype on the Frequency of Maternal Restraints of Infants

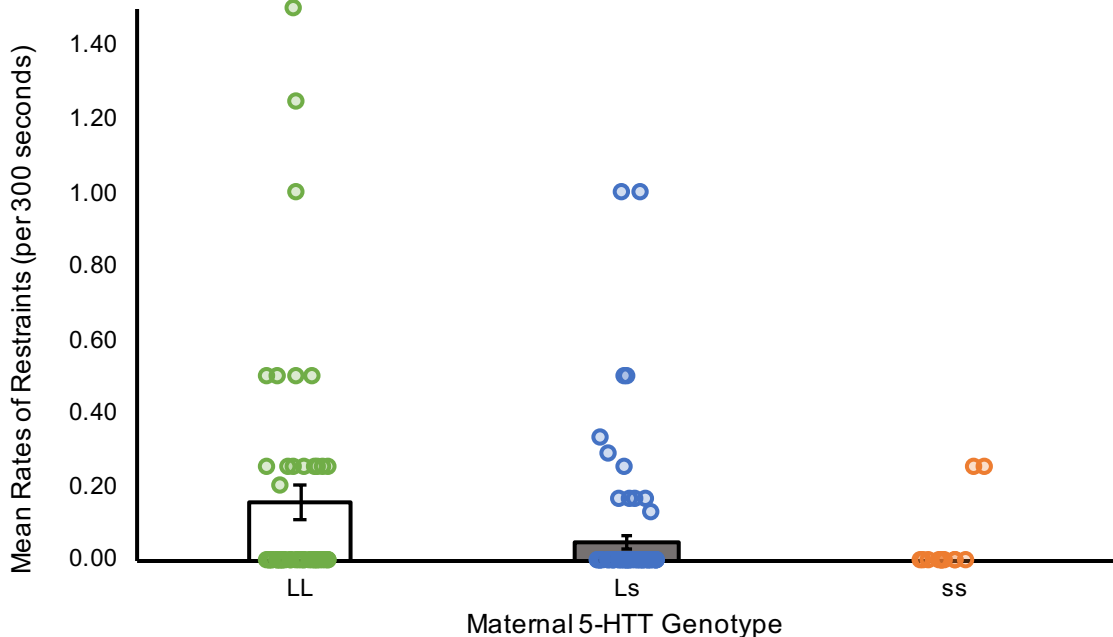

Supplement: S2 Fig — Plot depicts jittered individual maternal restraint data points, grouped by maternal 5-HTT genotype. When compared to heterozygous mothers and mothers that were homozygous for the s allele, mothers homozygous for the L allele exhibited the highest rates of maternal restraints (p = .02). Mothers that were homozygous for the L allele restraining their infants more, on average (p < .01), when compared to heterozygous mothers and mothers homozygous for the s allele (p < .03). Mothers that were homozygous for the s allele did not exhibit any restraints, indicating a robust effect with each addition of the s allele. White bars/green data points indicate mothers that were homozygous for the L allele, gray bars/blue data points indicate heterozygous mothers, and black bars/orange data points indicate mothers that were homozygous for the s allele. Data points are jittered to increase visibility. Error bars are standard errors. (PDF) [file pone.0281935.s002.pdf]
